# Glycemic Outcomes During Early Use of the MiniMed™ 780G Advanced Hybrid Closed-Loop System with Guardian™ 4 Sensor

**DOI:** 10.1089/dia.2023.0123

**Published:** 2023-08-23

**Authors:** Toni L. Cordero, Zheng Dai, Arcelia Arrieta, Fang Niu, Melissa Vella, John Shin, Andrew S. Rhinehart, Jennifer McVean, Scott W. Lee, Robert H. Slover, Gregory P. Forlenza, Dorothy I. Shulman, Rodica Pop-Busui, James R. Thrasher, Mark S. Kipnes, Mark P. Christiansen, Bruce A. Buckingham, Catherine Pihoker, Jennifer L. Sherr, Kevin B. Kaiserman, Robert A. Vigersky

**Affiliations:** ^1^ Medtronic, Northridge, California, USA.; ^2^ Medtronic International Trading Sàrl, Tolochenaz, Switzerland.; ^3^ Department of Endocrinology, Loma Linda University, Loma Linda, California, USA.; ^4^ Department of Pediatrics, Barbara Davis Center of Childhood Diabetes, Aurora, Colorado, USA.; ^5^ University of South Florida Diabetes and Endocrinology, Department of Pediatrics, Tampa, Florida, USA.; ^6^ Division of Metabolism, Endocrinology and Diabetes, University of Michigan, Ann Arbor, Michigan, USA.; ^7^ Arkansas Diabetes and Endocrinology Center, Little Rock, Arkansas, USA.; ^8^ Diabetes and Glandular Disease Clinic, San Antonio, Texas, USA.; ^9^ Diablo Clinical Research Center, Walnut Creek, California, USA.; ^10^ Stanford University School of Medicine, Department of Pediatric Endocrinology, Stanford, California, USA.; ^11^ Department of Pediatrics, University of Washington, Seattle, Washington, USA.; ^12^ Department of Pediatrics, Yale University School of Medicine, New Haven, Connecticut, USA.; ^13^ SoCal Diabetes, Torrance, California, USA.

**Keywords:** Type 1 diabetes, Advanced hybrid closed-loop, Glucose sensor, Non-adjunctive, Time-in-range, Real-world

## Abstract

**Background:**

Safety and significant improvement in overall glycated hemoglobin (A1C) and percentage of time spent in (TIR), below (TBR), and above (TAR) glucose range were demonstrated in the pivotal trial of adolescents and adults using the MiniMed™ advanced hybrid closed-loop (AHCL) system with the adjunctive, calibration-required Guardian™ Sensor 3. The present study evaluated early outcomes of continued access study (CAS) participants who transitioned from the pivotal trial investigational system to the approved MiniMed™ 780G system with the non-adjunctive, calibration-free Guardian™ 4 Sensor (MM780G+G4S). Study data were presented alongside those of real-world MM780G+G4S users from Europe, the Middle East, and Africa.

**Methods:**

The CAS participants (*N* = 109, aged 7–17 years and *N* = 67, aged >17 years) used the MM780G+G4S for 3 months and data of real-world MM780G+G4S system users (*N* = 10,204 aged ≤15 years and *N* = 26,099 aged >15 years) were uploaded from September 22, 2021 to December 02, 2022. At least 10 days of real-world continuous glucose monitoring (CGM) data were required for analyses. Glycemic metrics, delivered insulin and system use/interactions underwent descriptive analyses.

**Results:**

Time in AHCL and CGM use were >90% for all groups. AHCL exits averaged 0.1/day and there were few blood glucose measurements (BGMs) (0.8/day–1.0/day). Adults in both cohorts met most consensus recommendations for glycemic targets. Pediatric groups met recommendations for %TIR and %TBR, although not those for mean glucose variability and %TAR, possibly due to low use of recommended glucose target (100 mg/dL) and active insulin time (2 h) settings (28.4% in the CAS cohort and 9.4% in the real-world cohort). The CAS pediatric and adult A1C were 7.2% ± 0.7% and 6.8% ± 0.7%, respectively, and there were no serious adverse events.

**Conclusions:**

Early clinical use of the MM780G+G4S was safe and involved minimal BGMs and AHCL exits. Consistent with real-world pediatric and adult use, outcomes were associated with achievement of recommended glycemic targets.

Clinical Trial Registration number: NCT03959423

## Introduction

Continuous glucose monitoring (CGM) provides essential sensor glucose (SG) values at 1- or 5-min intervals,^1^ and allows visualization of current and impending hypoglycemic and hyperglycemic excursions. Recent systematic review and meta-analysis have shown that while adjunctive CGM technology associates with a greater reduction in glycated hemoglobin (A1C) and non-adjunctive CGM technology associates with a greater increase in percentage of time in range (%TIR), both similarly reduce the percentage of time spent below target SG range (%TBR) when compared with blood glucose measurement (BGM).^2^ The impact on %TBR is substantial, as nonsevere hypoglycemic events (NSHEs) in insulin-treated diabetes are common^3^ and hypoglycemia, in general, poses a significant economic^4,5^ and family burden^6,7^; with nocturnal NSHEs disrupting sleep, wellbeing, and work productivity.^8,9^

Automated insulin delivery (AID) systems with hybrid closed-loop (HCL) or advanced hybrid closed-loop (AHCL) algorithms modulate insulin delivery based on real-time CGM and a system-specific SG range or target to minimize hypoglycemia and improve %TIR.^10^ Several 6-month randomized controlled trials (RCTs) have demonstrated safe and clinically significant improvement in A1C,^11–14^ %TIR,^11–15^ and %TBR <70 mg/dL^11–15^ with different AID therapies; all of which comprised either adjunctive or non-adjunctive CGM technology components.

Real-world analyses of AID systems have supported many of the clinical trial findings and shown the widespread effectiveness of HCL^16–18^ and AHCL^19–22^ with either adjunctive or non-adjunctive CGM technology, and in pediatric and adult populations with T1D. The present study reports on early pediatric and adult glycemic outcomes during pivotal trial continued access study (CAS) and real-world use of the MiniMed™ 780G system with the Guardian™ 4 Sensor.

## Methods

### Pivotal trial continued access study cohort

The Safety Evaluation of the AHCL System in Type 1 Adult and Pediatric Subjects pivotal trial (NCT03959423) was a multicenter, single-arm, nonrandomized study that investigated safety, change in A1C, and the percentage of time spent in CGM ranges between baseline run-in (without AHCL) and study (with AHCL) in adolescent (aged 14–21 years) and adult (aged ≥22–75 years) participants. The investigational AHCL system included the MiniMed™ 670G insulin pump with version 4.0 algorithm, the adjunctive and calibration-required Guardian™ Sensor 3 (GS3) glucose sensor (Medtronic), Guardian™ Link 3 transmitter (Medtronic), and the CONTOUR^®^NEXT LINK 2.4 BG meter (Ascensia Diabetes Care, Parsippany, NJ). The primary safety and glycemic endpoint findings, in addition to protocol requirements, IRB-obtained approval, and criteria for study inclusion and exclusion, have been published.^23^

Participants who completed the pivotal trial could continue using the investigational system in a prospective continued access study (CAS). For those who entered the CAS, bloodwork for laboratory A1C was conducted at the end of the pivotal trial or at the start of the CAS period. Bloodwork for A1C was conducted, again, at 90-day follow-up visits. System data were also uploaded to CareLink™ Clinical software (Medtronic). Participants and/or their guardians were instructed to continue performing a BGM when prompted by the pump (e.g., for calibration) or when experiencing symptoms that did not align with their SG value. Investigational sites continued to report serious adverse events, including severe hypoglycemia and diabetic ketoacidosis (DKA), and system settings were adjusted at the discretion of investigators.

In May 2021, the MiniMed™ 780G system with Guardian™ 4 Sensor (G4S), Guardian™ Link 4 transmitter and the Accu-Chek^®^ Guide Link blood glucose meter (Roche Diabetes Care, Inc., Indianapolis, IN) received Conformité Européenne (CE) mark. The CE-marked system included the non-adjunctive indication for the G4S that replaces fingerstick BGMs for diabetes treatment decisions. Participants could transition from the investigational AHCL+GS3 system to the CE-marked system (MM780G+G4S) that included the same AHCL algorithm, an additional 110 mg/dL glucose target (GT) setting and Bluetooth™ low-energy connectivity.

### Real-world cohort

The real-world analyses included individuals (*N* = 36,303) who self-reported their T1D and their age as ≤15 years and >15 years, and who lived in Austria, Belgium, the Czech Republic, Denmark, Egypt, Finland, France, Germany, Great Britain, Iceland, Ireland, Israel, Italy, Luxembourg, Netherlands, Poland, Portugal, Qatar, Romania, Saudi Arabia, Slovenia, Slovakia, Sweden, Switzerland, South Africa, or the United Arab Emirates. From September 22, 2021, to December 2, 2022, MM780G+G4S system data were uploaded to CareLink™ Personal software (Medtronic) and ≥10 days of CGM data were required for analyses.

### Glycemic outcomes, insulin, and system use

For the CAS cohort, mean ± standard deviation (SD) of A1C was determined. For both the CAS and real-world cohorts, the mean ± SD of percentage of time spent in AHCL; percentage of CGM use, mean SG, coefficient of variation (CV) of SG, glucose management indicator (GMI), and percentage of time spent at SG ranges (i.e., <54 mg/dL, <70 mg/dL, 70–180 mg/dL, >180 mg/dL and >250 mg/dL) for the 24-h day and nighttime (12:00 AM to 05:59 AM) were assessed. The mean ± SD of total daily dose of insulin (TDD), total basal insulin, total bolus insulin, and system-initiated insulin (including Auto Correction insulin) were also determined. Additional analyses included system use/interactions involving the daily number of AHCL exits, user-initiated boluses, and BGMs for the 24-h day.

### Safety and descriptive analyses

While rates of severe hypoglycemia and DKA during the CAS were reported by investigators, the CareLink™ Personal platform does not capture these events. Thus, %TBR <54 mg/dL (i.e., level 2 hypoglycemia), which is considered clinically significant and deemed to require immediate attention,^24,25^ served as a safety endpoint proxy for the real-world analysis. For the CAS, severe hypoglycemia was defined as an event requiring the active assistance of another individual to administer carbohydrate, glucagon, or other resuscitative actions due to study participant altered consciousness. DKA was defined as a BG meter reading >250 mg/dL, arterial pH <7.3, bicarbonate <15 mEq/L, and moderate ketonuria or ketonemia requiring treatment in a medical facility. The mean or mean ± SD of glycemic metrics, delivered insulin, and system use/interactions were determined by age group for each cohort and underwent descriptive analyses.

## Results

### CAS participants and real-world users

Across 17 investigational sites, 109 children and adolescents (11.2 ± 2.5 years of age, *N* = 52 female) with diabetes duration of 6.6 ± 2.7 years, and 67 adults (45.4 ± 14.8 years of age, *N* = 36 female) with diabetes duration of 27.1 ± 12.8 years, transitioned from the investigational system to the MM780G+G4S system. The real-world cohort used the MM780G+G4S for a mean ± SD of 143.6 ± 100.7 days and included 10,204 (*N* = 4814 female) who self-reported an age ≤15 years (156.8 ± 103.9 system use days) and 26,099 (*N* = 14,215 female) who self-reported an age >15 years (138.5 ± 98.9 system use days).

### Glycemic outcomes, insulin, and system use

**FIG. 1.**
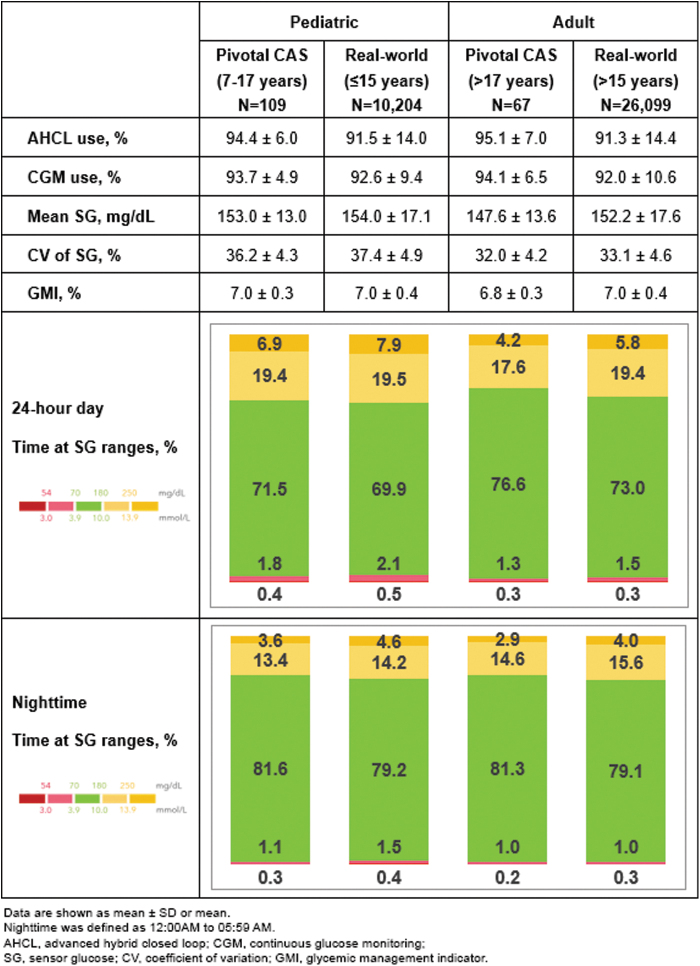
Glycemic outcomes during pediatric and adult pivotal trial CAS use and real-world use of the MiniMed™ 780G system with Guardian™ 4 Sensor. CAS, continued access study.

The time spent in AHCL, CGM use, and CGM outcomes for the 24-h day and percentage of time spent at SG ranges during the nighttime, for each age group during MM780G+G4S use are shown (Fig. 1). Insulin delivered, AHCL exits and the number of daily user-initiated boluses, and BGMs for both cohorts are listed (Table 1). For a qualitative comparison, some of the 24-h day data, in addition to the proportions meeting consensus recommended glycemic targets,^26^ during pivotal trial AHCL+GS3 use and real-world MM780G+GS3 use are provided, as a supplement (Supplementary Data S1).

**Table 1.** Daily Insulin Delivered and System Interactions During Pivotal Trial Continued Access Study and Real-World Use of the MiniMed™ 780G System with Guardian™ 4 Sensor

	Pediatric		Adult	
	Pivotal CAS (7–17 years)* N* = 109	Real-world (≤15 years)* N* = 10,204	Pivotal CAS (>17 years)* N* = 67	Real-world (>15 years)* N* = 26,099
TDD, U	52.0 ± 22.3	41.6 ± 23.6	59.9 ± 31.1	51.1 ± 25.6
Total basal, U	20.4 ± 9.2	16.1 ± 9.8	26.1 ± 14.5	21.6 ± 11.7
Total bolus, U	31.6 ± 13.7	25.5 ± 14.6	33.8 ± 18.1	29.5 ± 15.4
System-initiated insulin, U (%TDD)	30.4 ± 14.9 (58.0 ± 8.4)	24.2 ± 15.5 (56.8 ± 10.5)	35.4 ± 20.5 (58.1 ± 10.6)	30.4 ± 17.2 (59.1 ± 11.9)
Auto correction, U	10.1 ± 6.1	8.1 ± 6.3	9.2 ± 6.8	8.8 ± 6.6
Auto correction, %TDD (%Total Bolus)	18.7 ± 5.6 (31.3 ± 10.5)	18.4 ± 6.9 (30.6 ± 12.8)	14.7 ± 5.6 (26.8 ± 11.5)	16.6 ± 7.4 (29.9 ± 14.8)
User-initiated insulin, U (%TDD)	21.5 ± 9.5 (42.0 ± 8.4)	17.4 ± 10.3 (43.2 ± 10.5)	24.5 ± 13.5 (41.9 ± 10.6)	20.7 ± 11.9 (40.9 ± 11.9)
System interactions				
AHCL exits, *N*/day	0.1 ± 0.1	0.1 ± 0.2	0.1 ± 0.1	0.1 ± 0.1
User-initiated boluses, *N*/day	5.5 ± 2.0	5.7 ± 2.2	4.7 ± 1.7	4.9 ± 2.0
BGM, *N*/day	0.8 ± 0.5	1.0 ± 1.2	0.8 ± 0.9	0.8 ± 0.9

Data are shown as mean ± SD.

System-initiated insulin included programmed open-loop basal rates, Auto Basal and Auto Correction.

AHCL, advanced hybrid closed loop; BGM, blood glucose measurement; CAS, continued access study; SD, standard deviation; TDD, total daily dose of insulin.

### Safety

During the 3-month CAS, there were no serious adverse events, DKA or severe hypoglycemic events. For the real-world cohort, system users ≤15 years of age and >15 years of age spent a mean of 7.8 min/day (54.6 min/week) and 4.8 min/day (33 min/week), respectively, at %TBR <54 mg/dL.

## Discussion

In the present study, time in AHCL and CGM use were high for the pediatric and adult groups using the MM780G+G4S in the CAS and real-world cohorts. The CAS cohort demonstrated 3-month mean A1C that averaged 7.0% and had slightly lower mean SG, CV of SG, percentage of time spent above range (%TAR), and %TBR, with slightly increased TIR, relative to their respective age group in the real-world cohort. Adults met most consensus recommended glycemic targets for AID,^26^ which were further improved during the nighttime.

While the pediatric groups had very low exposure to hypoglycemia, they did not achieve consensus targets for mean SG, CV of SG, %TAR >180 mg/dL, and %TAR >250 mg/dL. Reasons for this may have been due to modifiable system settings. For instance, in the pediatric CAS cohort, only 28.4% (*N* = 31/109) used the lowest 100 mg/dL GT with the 2 h active insulin time (AIT) setting, for ≥10 days of CGM use. Even fewer used these settings in the pediatric real-world cohort (9.4% [*N* = 962/10,204]).

The Petrovski et al., 3-month RCT, recently reported on the impact of the 100 mg/dL GT (94% of participants) and 2 h AIT (80%–94% of participants) in individuals aged 12–18 years with T1D.^27^ It investigated simplified meal bolus announcements during MM780G+G4S use and observed a %TIR of 73.5% ± 6.7% for the simplified carb announcement group (*N* = 17) and a %TIR of 80.3% ± 7.4% for the precise carb announcement group (*N* = 17). The end of study %TAR >180 mg/dL were 19.0% ± 5.2% and 13.5% ± 5.9%, respectively; and %TAR >250 mg/dL were 5.7% ± 3.6% and 3.0% ± 2.4%, respectively.^27^ Interestingly, insulin-to-carb ratios (ICRs) were adjusted early in the study (no later than 2 weeks after system start) and were significantly reduced in both groups. In addition, a significantly lower percentage of mean Auto Correction insulin (15% of TDD and 22.6% of total bolus vs. 29% of TDD and 46% of total bolus [*P* = 0.003]) was observed for the precise carb announcement group. The same group had a significantly greater number of daily meal announcements (5.1 ± 1.1 vs. 3.7 ± 0.9 [*P* = 0.003]). Although only a small single-site study, glycemic outcomes improved for both groups who used MDI therapy at baseline, and there were no DKA or severe hypoglycemic events.

The Petrovski et al. findings and the present study pediatric outcomes, where Auto Correction insulin averaged 19% of TDD and 31% of total bolus, shed light on how ICR adjustments and specific modifiable settings inherent to AID therapy can help children and adolescents who may have difficulty managing hyperglycemia when compared to adults. As previously reported,^19,28^ the extent of delivered automated correction bolus can serve as a metric for guiding system settings and treatment decisions. This becomes even more important for T1D populations experiencing challenges with glycemia (e.g., high A1C and %TAR, or low %TIR),^28–31^ but for whom significant gains can be made with AID.^10,32^

The GS3 was the first glucose sensor approved for AID therapy.^33^ It demonstrated a mean absolute relative difference (MARD) of 10.6% ± 9.6% (11,664 paired points) in its pivotal trial^34^ and a MARD of 10.8% ± 9.0% (3710 paired points) in the MiniMed™ 670G system pivotal trial.^35^ The GS3 with calibration-free algorithm (i.e., G4S) demonstrated a MARD of 10.8% ± 9.3% (18,423 paired points) in its pivotal trial^36^ and has been used in the clinical^37,38^ and real-world^39^ setting since 2021. Similar to the study phase-period of the investigational AHCL pivotal trial with GS3, there were no severe hypoglycemic or DKA events during CAS MM780G+G4S use in the present study.

Regarding real-world safety of the MM780G+G4S, the 7.8 min/day and 4.8 min/day at %TBR <54 mg/dL for younger and older users, respectively, indicate very low exposure to level 2 hypoglycemia. A recent modeling analysis based on pivotal trial participants, real-world system users and simulated virtual patients transitioning from GS3 to G4S ascertained the clinical impact of that transition.^40^ Real-world comparisons (*N* = 1335 users) before and after transition demonstrated a %TBR <54 mg/dL that decreased by 0.4% (6 min/day), indicating a very low risk of serious hypoglycemia.

In addition to the MM780G+G4S glycemic benefits observed in the CAS and real-world cohorts, T1D management burden was reduced. Supplementary data show that, compared to pivotal trial AHCL+GS3 use, there were slightly fewer closed-loop exits during MM780G+G4S use by both age groups. In addition, there were markedly fewer daily BGMs by the CAS pediatric group (0.8 ± 0.5/day vs. 4.2 ± 1.2/day) and older group (0.8 ± 0.9/day vs. 4.0 ± 1.0/day) when using MM780G+G4S. Similar reductions in daily BGMs for the real-world pediatric group (1.0 ± 1.0/day vs. 3.4 ± 1.0/day) and real-world adult group (0.8 ± 0.9/day and 3.2 ± 0.9/day) were observed during MM780G system use with G4S versus GS3.

A limitation of the present study is that CAS data were collected prospectively and real-world data were retrospectively assessed, which prevented statistical comparative analyses. The study population difference added to this, as the CAS cohort involved clinical trial-enrolled participants with known medical history, AID system experience, follow-up, and moderate glycemic control at baseline (A1C of 7.5% ± 0.8%). In contrast, and while representing potentially generalized outcomes, the larger real-world cohort from multiple countries had no known demographic, medical history, or clinical laboratory follow-up information. Study strengths include timely access to MM780G+G4S for the CAS cohort, in addition to pediatric and adult real-world MM780G system users having automatic data uploading capability through the MiniMed™ smartphone application. This allowed visualization of clinical outcomes and system interactions alongside those of real-world system users.

## Conclusions

Early 3-month use of the MiniMed™ 780G system with the Guardian™ 4 Sensor in a clinical setting was safe. Most adults in the clinical and real-world setting achieved consensus glycemic targets, while many children and adolescents in both settings met recommended GMI, %TIR, and %TBR targets.

## Supplementary Material

Supplemental data
