# Supplementary figures and images for: Glycemic Outcomes During Early Use of the MiniMed™ 780G Advanced Hybrid Closed-Loop System with Guardian™ 4 Sensor
Source: Diabetes Technol Ther. 2023 Aug 23;25(9):652–8. doi: 10.1089/dia.2023.0123 (PMC10460682; doi:10.1089/dia.2023.0123)

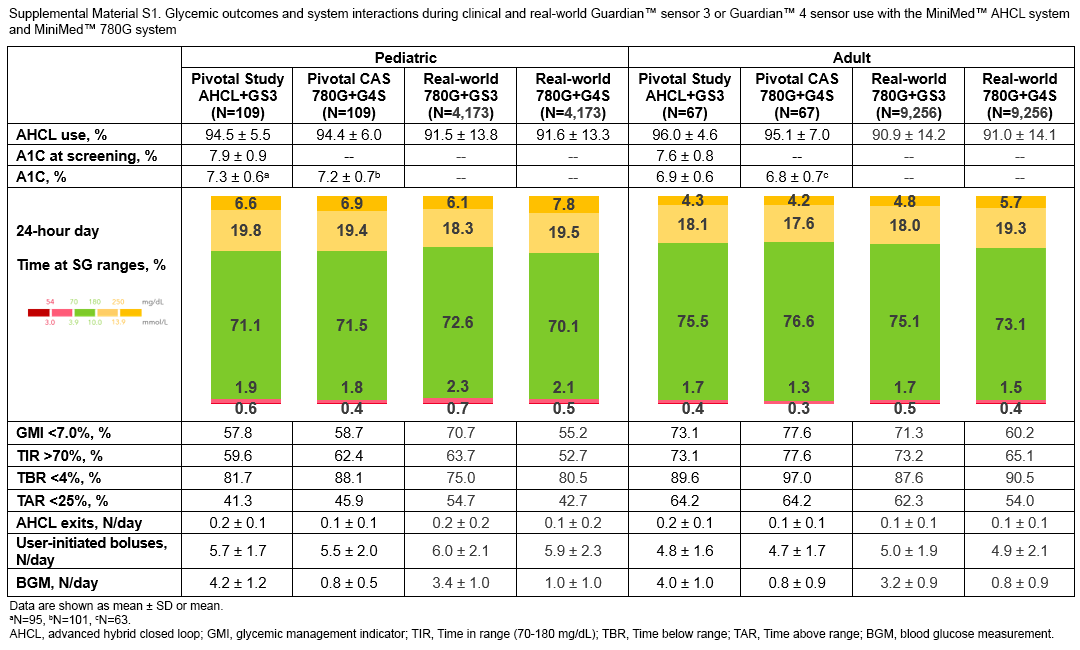

Supplement: Supplemental data [file Suppl_DataS1.tif]
